# Evaluation of the Ecological Effects of Ecological Restoration Programs: A Case Study of the Sloping Land Conversion Program on the Loess Plateau, China

**DOI:** 10.3390/ijerph19137841

**Published:** 2022-06-26

**Authors:** Yuanjie Deng, Lei Jia, Yajun Guo, Hua Li, Shunbo Yao, Liqi Chu, Weinan Lu, Mengyang Hou, Binbin Mo, Yameng Wang, Haiyu Yang, Tongyue Zhang

**Affiliations:** 1 College of Economics and Management, Northwest A&F University, Yangling 712100, China; dengyuanjie@nwafu.edu.cn (Y.D.); jia881810@nwafu.edu.cn (L.J.); guoyajun71@nwafu.edu.cn (Y.G.); lihua7485@nwafu.edu.cn (H.L.); cnclq@nwafu.edu.cn (L.C.); luweinan@nwafu.edu.cn (W.L.); mobinbin@nwafu.edu.cn (B.M.); wym@nwafu.edu.cn (Y.W.); yanghaiyu@nwafu.edu.cn (H.Y.); tongyue.zhang@nwafu.edu.cn (T.Z.); 2 Center for Resource Economics and Environment Management, Northwest A&F University, Yangling 712100, China; 3 School of Economics, Hebei University, Baoding 071000, China; houmengyang@hbu.edu.cn; 4 Research Center of Resources Utilization and Environmental Conservation, Baoding 071000, China

**Keywords:** ecological restoration programs, ecological effects, Loess Plateau, Sloping Land Conversion Program (SLCP)

## Abstract

The Sloping Land Conversion Program (SLCP) is the largest ecological restoration program in the world. Evaluating the ecological effects of the SLCP not only provides a scientific basis for China to improve the SLCP but also provides a reference for other countries in the world to evaluate the ecological effects of ecological restoration programs being implemented or to be implemented. To this end, we took the Loess Plateau, the core area for the implementation of the SLCP, as an example and, based on multi-source remote sensing data and GIS technology, we conducted a comprehensive evaluation of the ecological effects of the implementation of the SLCP on the Loess Plateau. The results showed that, first, from 2000 to 2018, a total of 12,372.05 km^2^ of cultivated land was converted into forest land and grassland on the Loess Plateau, and this contributed to an increase in vegetation cover from 45.09% in 2000 to 64.15% in 2018, and a decrease in the soil erosion modulus from 26.41 t·hm^−2^·yr^−1^ in 2000 to 17.92 t·hm^−2^·yr^−1^ in 2018. Second, the 6–25° slope range is the core area of the Loess Plateau for implementation of the SLCP. In this range, the area of cultivated land converted into forest land and grassland accounts for 60.16% of the total area of transferred cultivated land. As a result, the 6–25° slope range has become the most significant area for improving vegetation cover and reducing the soil erosion intensity, and it is mainly concentrated in the southwestern, central and central-eastern hilly and gully areas of the Loess Plateau. Third, from 2000 to 2018, the climate of the Loess Plateau tended to be warm and humid and was conducive to the implementation of the SLCP. Among these factors, precipitation is the dominant factor in determining the spatial distribution of vegetation on the Loess Plateau, and the increase in precipitation is also the main reason for the promotion of vegetation growth. Fourthly, from 2000 to 2018, the ecological environment of the Loess Plateau was significantly improved as a result of the combined effects of the implementation of the SLCP and climate warming and humidification, but the primary reason is still the implementation of the SLCP.

## 1. Introduction

Over the past 50 years, with rapid global economic development and population growth, human demand for natural resources has far exceeded the Earth’s productive supply, resulting in the degradation of 60% of the Earth’s ecosystems [1,2,3]. A series of ecological and environmental problems caused by ecosystem degradation, such as forest decline, flooding, global warming, land degradation and species extinction, have posed a great threat to human social development [4,5]. Since the implementation of the reform and opening-up policy in the late 1970s, China’s rapid socio-economic development has exacerbated the abovementioned ecological and environmental problems, to the detriment of socio-economic development. In response to increasingly serious ecological and environmental problems, the Chinese government has implemented major ecological restoration programs nationwide since the beginning of the 21st century, such as the Natural Forest Conservation Program (NFCP), the Three North Shelterbelt Development Program (TNSP) and the Sloping Land Conversion Program (SLCP) [6,7,8].

The decision to implement the SLCP was prompted by the massive floods that occurred in the Yangtze, Songhua and Neng River basins in China at the end of the 20th century, which made the Chinese government aware of the serious dangers of soil erosion [9,10]. The core objective of the SLCP is to curb soil erosion by stopping cultivation on sloping land that is prone to erosion and then planting trees according to the principle of having appropriate trees on appropriate land [11,12]. The SLCP was piloted in Sichuan, Shaanxi and Gansu Provinces in 1999 and fully launched in all of China in 2002, and then a new round of the SLCP was launched in 2014. As of 2018, the Chinese government has invested a total of about CNY 500 billion, with a total of more than 500 million mu of cultivated land returned to forests, and more than 150 million farmers have directly benefited [13,14]. Therefore, the SLCP has become the largest ecological restoration program in China and even in the world in terms of four characteristics: the largest capital investment, the largest scale of construction, the strongest policy and the highest degree of people’s participation [13,15,16]. As an ecological restoration program, evaluations of the ecological effects of the SLCP have always been an important issue of concern to policymakers and scholars.

In evaluating the ecological effects of the SLCP, previous studies have focused on three aspects. First, regarding the impact of the SLCP on land use change, scholars have found that the land use structure of the study area has changed significantly due to the implementation of the SLCP, which is characterized by the reduction of cultivated sloping land and an increase in forest land and grassland [17,18,19,20,21]. The second aspect is the impact of the SLCP on the vegetation cover. Scholars have found that the vegetation cover of the study area was significantly improved after the implementation of the SLCP [22,23,24,25,26,27]. Meanwhile, scholars have further studied the specific contribution of the SLCP to improvements in vegetation cover. For example, Wang et al. [28] concluded that the contribution of the SLCP to the increase in vegetation cover in China was about 26.33%, and Zheng et al. [29] concluded that the contribution of the SLCP to the increase of vegetation cover on the Loess Plateau was about 42.35%. Third, regarding the impact of the SLCP on ecosystem services, scholars have found that the SLCP has significantly changed the supply of ecosystem services such as water conservation [30,31,32], soil retention [14,23,33,34], carbon storage [35,36,37] and biodiversity [38,39,40] in the study area. Meanwhile, scholars have further investigated the impact of the SLCP on the trade-offs and synergies between different ecosystem services. For example, Wang et al. [41] concluded that the implementation of the SLCP in northwestern Yunnan, China, increased soil retention but led to a decrease in water yield, with a trade-off between soil retention and water yield. He et al. [42] concluded that water yield and food supply on the Loess Plateau have synergy, but a trade-off between water yield and habitat quality is necessary. That is, the SLCP has improved the habitat quality of the Loess Plateau but has led to a decrease in water yield and food supply.

In summary, previous studies have evaluated the ecological effects of the SLCP from the above three research perspectives and have made important contributions to policymakers’ in-depth understanding of the SLCP. However, while most of the previous studies have focused on evaluating the ecological effects of the SLCP from one research perspective, a comprehensive and systematic evaluation of the ecological effects of the SLCP integrating the above three research perspectives is lacking. The essence of the SLCP is to achieve ecological restoration by adjusting the land use structure. That is to say, the cultivation of steep slopes prone to soil erosion is stopped, and then vegetation cover and ecosystem services are increased by converting the slopes into forest land and grassland [11,12]. Therefore, the SLCP will essentially only have an impact on land use change but will then indirectly affect vegetation cover and ecosystem services through the resulting land use change [43,44]. Land use change is the basis for changes in vegetation cover and ecosystem services, and there is a coupling relationship among these three aspects [45,46]. If we evaluate the ecological effect of the SLCP from only one research perspective, it will make the evaluation of the ecological effects of the SLCP biased, which makes it difficult to provide an accurate decision basis for improving the SLCP.

Our research goes beyond the limitations of the previous studies. First, we took the Loess Plateau, the core area for the implementation of the SLCP, as an example and used GIS technology to make a comprehensive evaluation of the changes in land use, vegetation cover and ecosystem services after the implementation of the SLCP on the Loess Plateau based on multi-source remote sensing data. Second, based on land use changes, we identified the core areas of the Loess Plateau for implementing the SLCP and further analyzed the specific responses of vegetation cover and ecosystem service changes to land use changes. Finally, we analyzed the influence of climate factors on the effects of the SLCP on the Loess Plateau.

## 2. Materials and Methods

### 2.1. Study Area

**Figure 1:**
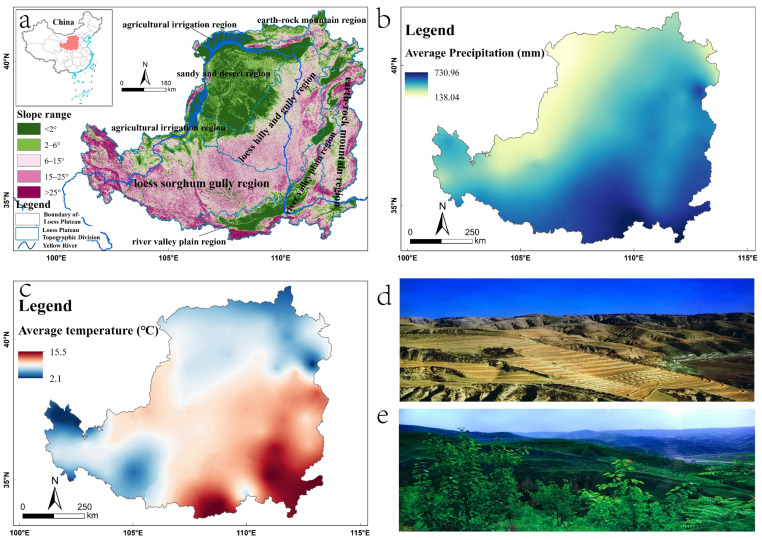
(**a**) Map of the Loess Plateau’s slope range and topographic division, (**b**) average precipitation distribution on the Loess Plateau from 2000 to 2018; (**c**) average temperature distribution across the Loess Plateau from 2000 to 2018; (**d**) Loess Plateau before the implementation of the SLCP; (**e**) Loess Plateau after the implementation of the SLCP.

The Loess Plateau is located in the central north of China (33°43′ N~41°16′ N, 100°54′ E~114°33′ E), involving the middle and upper reaches of the Yellow River basin, covering an area of about 650,000 km^2^. The overall topography of the Loess Plateau is high in the northwest and low in the southeast, with an elevation range of 82–5210 m. The Loess Plateau has a variety of landform types and is usually divided into six large zones [47] according to landform type (Figure 1a). The average annual precipitation of the Loess Plateau is 138–730 mm, mostly concentrated in July–September, with heavier precipitation [48] having a spatially decreasing distribution from the southeast to the northwest (Figure 1b). The annual average temperature of the Loess Plateau is 2.1–15.5 °C, and its horizontal and vertical distribution varies greatly (Figure 1c). Since the 1970s, as China’s industrialization and urbanization have accelerated, the Loess Plateau has suffered from excessive deforestation and grazing, resulting in a rapid reduction in surface vegetation, increasingly serious land degradation and a deteriorating ecological environment. At the beginning of the 21st century, in order to improve the deteriorating ecological environment of the Loess Plateau, the Chinese government began to implement the SLCP on the plateau. Through the conversion of large areas of cultivated land to forest land and grassland (Figure 1d), the ecological environment of the Loess Plateau has been significantly improved, vegetation cover has increased, and the serious situation of soil erosion has been effectively curbed [49].

### 2.2. Methodology

This study will integrate three research perspectives, namely land use, vegetation cover and ecosystem services, in order to comprehensively evaluate the ecological effects of the SLCP. The specific research methods are described below.

#### 2.2.1. Land Use Transfer Matrix

The land use transfer matrix not only portrays the structural characteristics and change process of land use but also reflects the direction of change among land use types [50]. Using the land use type area as a vector in the transfer matrix can reveal the spatial and temporal transformation process of regional land use types within a certain time interval. The calculation formula is as follows:

(1) $$S_{ij}=\left[\begin{array}{cccc} S_{11} & S_{12} & \cdots & S_{1n}\\ S_{21} & S_{22} & \cdots & S_{2n}\\ \cdots & \cdots & \cdots & \cdots \\ S_{n1} & S_{n2} & \cdots & S_{nn} \end{array}\right]$$

where *S* represents the land use area; *i* and *j* represent the land use types at the beginning and end of the study period, respectively; *n* is the total number of land use types; and *S_ij_* is the area of land use transferred from type *i* to type *j* during the study period.

#### 2.2.2. Vegetation Fraction Cover

Based on *NDVI* data, a pixel dichotomy model [51] was selected to calculate the vegetation fraction cover (*VFC*). The formula is as follows:

(2) $$VFC=\left(NDVI-NDVI_{s}\right)/\left(NDVI_{v}-NDVI_{s}\right)$$

where *VFC* is the vegetation fraction cover (%); *NDVI* is the *NDVI* value of any image element; *NDVI_s_* is the *NDVI* value of a pure soil image element, which is theoretically close to 0; and *NDVI_v_* is the *NDVI* value of a pure vegetation image element, which is theoretically close to 1. The upper and lower thresholds of *NDVI* were intercepted using a 0.5% confidence level, the 0.5% area with the largest *NDVI* value was averaged to obtain *NDVI_v_*, and the 0.5% area with the smallest value was averaged to obtain *NDVI_s_*. With reference to previous studies [52], we classified the *VFC* of the Loess Plateau into the following four classes: low *VFC* (*VFC* < 25%), medium-low *VFC* (25% ≤ *VFC* < 50%), medium-high *VFC* (50% ≤ *VFC* < 75%) and high *VFC* (*VFC* ≥ 75%).

#### 2.2.3. Trend Analysis of VFC

The time series of *VFC* for each pixel from 2000 to 2018 in the Loess Plateau were linearly fitted to obtain the trend slopes. The trend of each pixel value for a certain time series can be simulated using univariate linear regression [53]. The equation for this is as follows:

(3) $$\mathrm{slope}\;=\frac{n\times \sum_{i=1}^{n}\left(i\times f_{i}\right)-\left(\sum_{i=1}^{n}i\right)\left(\sum_{i=1}^{n}f_{i}\right)}{n\times \sum_{i=1}^{n}i^{2}-{\left(\sum_{i=1}^{n}i\right)}^{2}}$$

where *n* is the number of years, which is 18 in this study; *f_i_* represents the pixel value of the *VFC* of the *i*th year. The slope is the trend of *VFC* from 2000 to 2018. A slope of >0 indicates an increasing trend, while a slope of <0 represents a decreasing trend during the 18-year period. By referring to previous studies [54,55], the *VFC* trends were classified as significant degradation (−0.055 < slope < −0.003), slight degradation (−0.003 ≤ slope < 0), slight improvement (0 ≤ slope < 0.01) and significant improvement (0.01 ≤ slope < 0.06).

#### 2.2.4. Soil Loss

In this study, the soil loss (*SL*) was estimated using the Revised Universal Soil Loss Equation (RUSLE) as follows [56]:

(4) SL=R×K×LS×C×P

where *SL* is the amount of annual soil loss (t·hm^−2^·yr^−^^1^); the detailed calculation of each factor is as follows.

(1) Rainfall erosion factor (*R*)

The rainfall erosion factor (*R*) is based on the empirical formula proposed by Wischmeier [57]:

(5) $$R=\sum_{i=1}^{12}1.735\times 10^{\left(1.5\mathrm{lg}\frac{p_{i}{}^{2}}{p}-0.8188\right)}\times 17.02$$

where *R* is rainfall erosivity (MJ·mm·ha^−1^·h^−1^ ·yr^−1^); *P_i_* is the monthly rainfall (mm); and *p* is the annual rainfall (mm). The value 17.02 is the conversion factor from the customary U.S. units to SI units.

(2) Soil erosive factor (*K*)

The soil erosive factor (*K*) was calculated by the erosion/productivity impact calculator (EPIC) model based on the soil texture [58]:

(6) $$\begin{array}{cc} K= & 0.1317\times \left\{0.2+0.3\times \exp\left[-0.0256SAN\left(1-\frac{SIL}{100}\right)\right]\right\}{\left[\frac{SIL}{CLA+SIL}\right]}^{0.3}\times \\ & \left\{1-0.25\times \frac{C}{C+\exp(3.72-2.95C)}\right\}\left\{1-0.7\times \frac{SN_{1}}{SN_{1}+\exp(22.95SN_{1}-5.51)}\right\} \end{array}$$

where *K* is the soil erodibility factor (t· h ·MJ^−1^· mm^−1^); *SAN*, *SIL* and *CLA* are the sand fraction (%), the silt fraction (%) and the clay fraction (%), respectively; *C* is the soil organic carbon content (%); and *SNI* is equal to 1–*SAN*/100. The value 0.1317 is the conversion factor from the customary U.S. units to SI units.

(3) Slope length and steepness factor (LS)

The slope threshold required to calculate the LS factor was used in the Loess Plateau [59].

(7) $$\begin{array}{l} L={\left(\lambda /22.13\right)}^{m}\\ S=\{\begin{array}{c} 10.8\sin\theta +0.03\theta <9\%\\ 16.8\sin\theta -0.50\theta \geq 9\% \end{array}\\ m=\beta /(1+\beta )\\ \beta =\sin\theta /[3{\left(\sin\theta \right)}^{0.8}+0.56] \end{array}$$

where *L* is the slope length factor, *S* is the slope steepness factor, *λ* is the horizontal projection slope length, *m* is the variable slope length exponent, *β* is a factor that varies with slope gradient, and *θ* is the slope angle (%).

(4) Vegetation cover and management factor (*C*)

*C* is the vegetation cover and management factor, which is dimensionless and obtained by using the regression equation between *VFC* and the *C* factor established by Cai et al. [60].

(8) $$C=\{\begin{array}{lll} 1 & & VFC=0\\ 0.6508-0.3436\mathrm{lg}VFC & & 0<VFC\leq 78.3\%\\ 0 & & VFC>78.3\% \end{array}$$

(5) Erosion control practice factor (*p*)

The erosion control practice factor *p* is dimensionless and is set according to the parameters described in publications relevant to the Chinese Loess Plateau [61]. This study defined the value of *p* for cultivated land, forest land, grassland as 0.31, 0.05 and 0.16, respectively. For the land use types of water, construction land and unused land, *p* equals 1.

According to the Technological Standard of Soil and Water Conservation SL 190-2007, issued by the Ministry of Water Resources of China [62], the quantitative output of the estimated soil loss was divided into six ordinal classes. The six erosion intensity classes are specified as follows: very slight (10 t·hm^−2^·yr^−1^ < soil erosion), slight (10 t·hm^−2^·yr^−1^ ≤ soil erosion < 25 t·hm^−2^·yr^−1^), moderate (25 t·hm^−2^·yr^−1^ ≤ soil erosion < 50 t·hm^−2^·yr^−1^), severe (50 t·hm^−2^·yr^−1^ ≤ soil erosion < 80 t·hm^−2^·yr^−1^), very severe (80 t·hm^−2^·yr^−1^ ≤ soil erosion < 150 t·hm^−2^·yr^−1^) and extremely severe (soil erosion ≥ 150 t·hm^−2^·yr^−1^).

### 2.3. Data Sources

Various remote sensing data such as land use, meteorological and normalized difference vegetation index (*NDVI*) data were used in this study. The specific sources and details of the data are listed below.

The land use data were obtained from the Data Center for Resources and Environmental Science, Chinese Academy of Sciences, and included two periods, 2000 and 2018, with a spatial resolution of 30 m (https://www.resdc.cn/ (accessed on 5 May 2022)). The dataset was based on 2000 Landsat7 ETM+ and 2018 Landsat8 OLS_TIRS remote sensing images, generated by manual visual interpretation and later corrected for errors through field verification. The final land use data accuracy can reach more than 93% [63,64]. According to the classification criteria of land use types in the project of “Remote Sensing Macro Survey and Dynamic Research on China’s Resources and Environment” [65], the land use types of the Loess Plateau are classified into six categories: cultivated land, forest land, grassland, water body, built-up land and bare land.

The meteorological data, including temperature and precipitation, were derived from the monthly value dataset of Chinese ground climate information provided by the China Meteorological Data Center (http://data.cma.cn/ (accessed on 6 September 2020)), spanning the period of 2000–2018. Using the meteorological station data obtained, the kriging interpolation method in the ArcGIS 10.7 software (ArcGIS software from Esri, CA, USA) was applied to spatially interpolate the temperature and precipitation, and finally the temperature and rainfall raster data of Loess Plateau were obtained.

The MODIS *NDVI* data were obtained from the MOD13A3 product provided by NASA with a spatial resolution of 1 km and a temporal resolution of 30 days (https://ladsweb.modaps.eosdis.nasa.gov/search/ (accessed on 10 April 2021)). In the processing of the data, firstly, the MODIS Reprojection Tool (MRT) was used for format and projection conversion, banding and mosaic processing. Secondly, the *NDVI* data of the study area were obtained by clipping the Loess Plateau boundary. Finally, the maximum value composite (MVC) was used to exclude sources of interference such as clouds and the atmosphere to obtain the *NDVI* data of the Loess Plateau from 2000 to 2018.

The DEM data were obtained from the SRTM product provided by OpenTopography (https://portal.opentopography.org (accessed on 5 March 2021)), which has a spatial resolution of 90 m.

Soil data were obtained from the Harmonized World Soil Database (HWSD) constructed by the Food and Agriculture Organization of the United Nations (FAO) and the International Institute for Applied Systems Analysis (IIASA) (http://webarchive.iiasa.ac.at/Research/LUC/External-World-soil-database (accessed on 25 August 2019)). The spatial resolution of the soil data is 1 km.

The basic geographic information includes the boundary of the Loess Plateau and its geomorphological subdivision boundaries, derived from the Loess Plateau SubCenter, National Earth System Science Data Center, National Science and Technology Infrastructure of China (http://loess.geodata.cn (accessed on 1 July 2019)).

## 3. Results

### 3.1. Land Use Change

**Figure 2:**
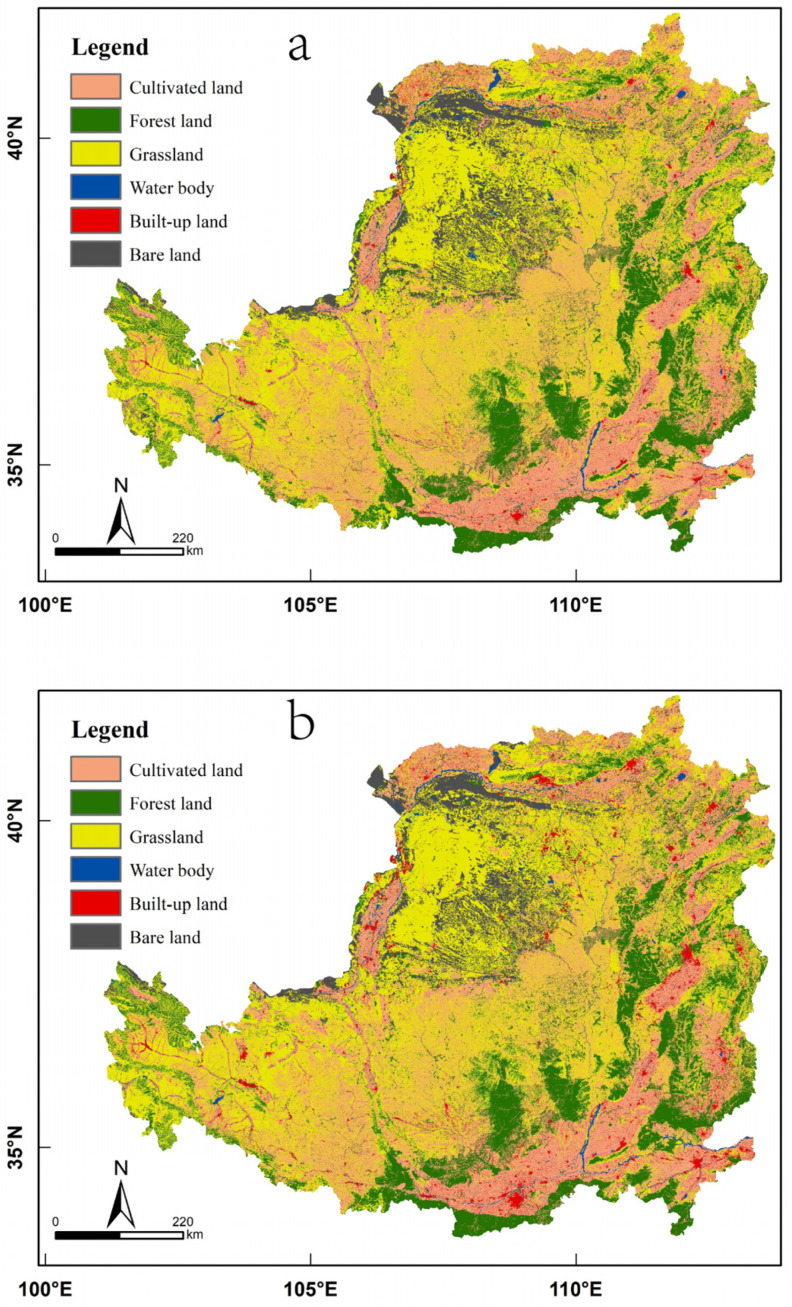
Land use map of the Loess Plateau in (**a**) 2000 and (**b**) 2018.

As shown by the land use structure (Table 1) and its spatial distribution (Figure 2) in the Loess Plateau from 2000 to 2018, the land use of the Loess Plateau is dominated by grassland, which accounts for more than 39% of the total area, mainly in the western, central and northern regions of the Loess Plateau. The second most dominant type is cultivated land, accounting for more than 31% of the total area, mainly in the river valley plain area in the south and east of the Loess Plateau and the irrigated agricultural area in the north. Forest land accounts for more than 16% of the total area and is mainly distributed in the southern, central and eastern mountainous areas of the Loess Plateau. Bare land accounts for more than 6% of the total area, mainly in the sandy and desert areas in the northwestern part of the Loess Plateau. Built-up land accounts for more than 2% of the total area, and its spatial distribution is basically consistent with the spatial distribution of cultivated land. The water body category has the least area, accounting for just over 1% of the total area of the region.

Further analysis of the changes in land use types showed that cultivated land, grassland, bare land and water bodies have shown decreasing trends, while forest land and built-up land have shown increasing trends.

Detailed information on the decreases in the areas of land use types in the Loess Plateau from 2000–2018 can be described as follows.

**Figure 3:**
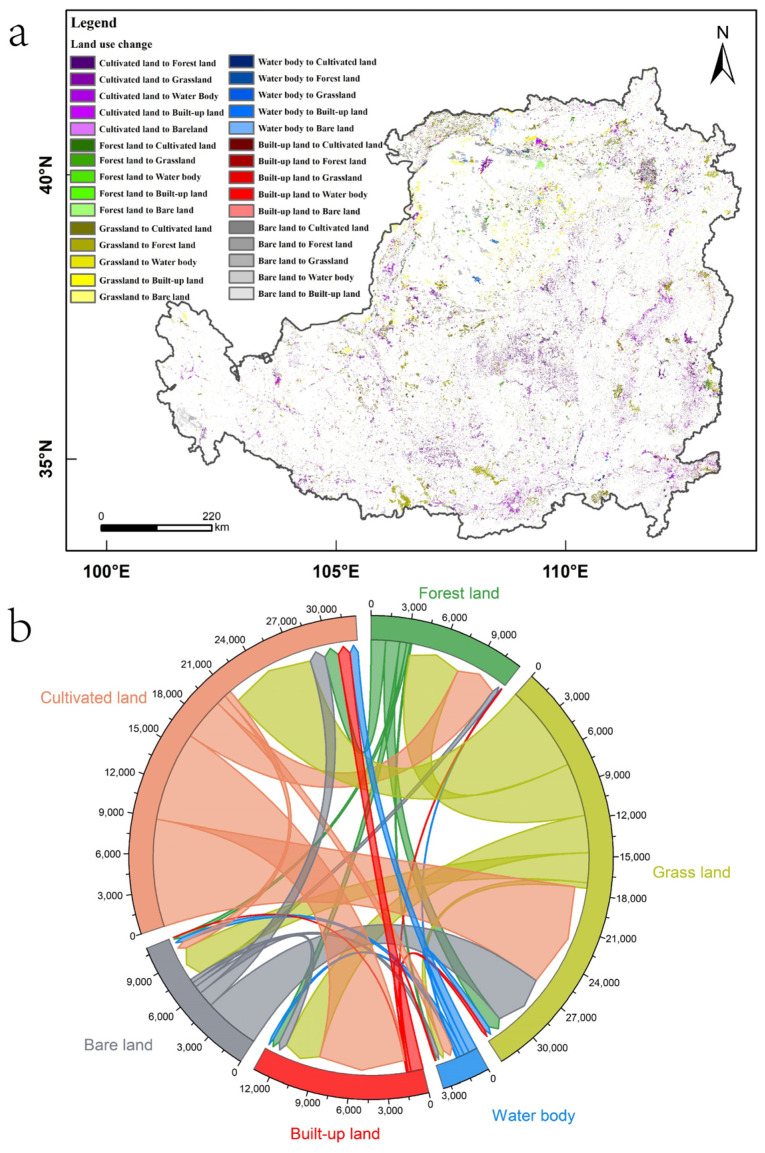
(**a**) Spatial patterns of land use change on the Loess Plateau during 2000–2018. (**b**) Land use quantity transfer chord diagram of the Loess Plateau during 2000–2018.

The cultivated land area decreased from 217,365.31 km^2^ in 2000 to 207,595.62 km^2^ in 2018, a total decrease of 9769.68 km^2^ in 18 years, with an average annual change rate of −0.25% (Table 1). According to the results of the transfer matrix, in terms of the number of transfers (Figure 3b), cultivated land was mainly transferred to grassland, built-up land and forest land, accounting for 41.90%, 35.10% and 16.53% of the total reduction, respectively. In terms of the spatial distribution of the transfers (Figure 3a), the transformation of cultivated land into grassland and forest land was mainly concentrated in the eastern part of the loess sorghum gully region, the southern and northern part of the loess hilly gully region and some parts of the earth–rocky mountain region. The conversion of cultivated land into built-up land was mainly concentrated in the river valley plain region and the irrigation agricultural region.

The grassland area decreased from 258,855.74 km^2^ in 2000 to 256,568.11 km^2^ in 2018, with a total decrease of 2287.63 km^2^ in 18 years and an average annual change rate of 0.05% (Table 1). According to the results of the transfer matrix, in terms of the number of transfers (Figure 3b), grassland was mainly transferred to cultivated land, forest land and built-up land, with the transferred area accounting for 35.67%, 34.70% and 17.21% of the total transferred area, respectively. In terms of the spatial distribution of the transfers (Figure 3a), the transformation of grassland into cultivated land was mainly concentrated in the irrigated agricultural region and the northern part of the loess sorghum gully region; the transformation of grassland into forest land was mainly concentrated in the eastern part of the sand and desert region, the southern part of the loess hilly gully region and the northern and southern part of the loess sorghum gully region; and the transformation of grassland into built-up land was mainly concentrated in the agricultural and irrigation region, the northeastern part of the sand and desert region and the northwestern part of the loess hilly gully region.

The area of bare land decreased from 43,300.14 km^2^ in 2000 to 39,769.35 km^2^ in 2018, a total decrease of 3530.79 km^2^ in 18 years, with an average annual change rate of 0.45% (Table 1). According to the results of the transfer matrix, in terms of the number of transfers (Figure 3b), most transferred bare land was transformed into grassland or cultivated land, with the transferred areas of those types accounting for 63.18% and 18.04% of the total transferred area, respectively. In terms of the spatial distribution of the transfers (Figure 3a), the transformation of bare land into grassland was mainly concentrated in the sand and desert region, and the transformation into cultivated land was mainly concentrated in the irrigation agricultural region.

The water body area changed little in 2000–2018, with an overall fluctuating downward trend, decreasing by only 58.40 km^2^ in 18 years (Table 1).

Detailed information on the increase in the areas of land use types in the Loess Plateau from 2000 to 2018 can be described as follows.

The forest land area increased from 104,521.47 km^2^ in 2000 to 109,614.19 km^2^ in 2018, with a total increase of 5092.72 km^2^ in 18 years and an average annual change rate of 0.27% (Table 1). According to the results of the transfer matrix, in terms of the number of transfers (Figure 3b), the increase in forest land mainly came from grassland and cultivated land, which accounted for 52.21% and 41.47% of the increase in forest land area, respectively. In terms of the spatial distribution of the transfers (Figure 3a), the increase in forest land is consistent with the spatial distribution of the transfers from cultivated to forest land and from grassland to forest land described above.

The area of built-up land increased from 16,072.09 km^2^ in 2000 to 26,626.24 km^2^ in 2018, with a total increase of 10,554.15 km^2^ in 18 years and an average annual change rate of 3.65% (Table 1). According to the results of the transfer matrix, in terms of the number of transfers (Figure 3b), the increase in built-up land mainly came from grassland and cultivated land, and the transfers from grassland and cultivated land to built-up land accounted for 66.37% and 15.50% of the increase in the built-up land area, respectively. In terms of the spatial distribution of the transfers (Figure 3a), the increase in built-up land is consistent with the spatial distribution of the transfer of grassland to built-up land and the transfer of cultivated land to built-up land mentioned above.

### 3.2. VFC Change

**Figure 4:**
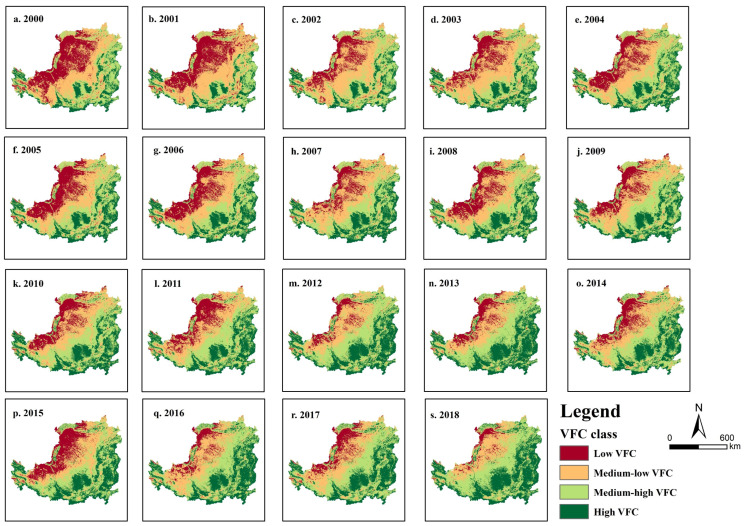
Spatial distribution of *VFC* in the Loess Plateau from 2000 to2018.

From the spatial distribution of *VFC* on the Loess Plateau (Figure 4), it can be concluded that the high *VFC* areas are mainly distributed in the southern, eastern and western regions. The medium-high *VFC* areas are mainly located in the southern, central and northeastern regions. The low-medium *VFC* areas are mainly located in the northern, northwestern and western regions, and the low *VFC* areas are mainly located in the northwestern and central-western regions. Overall, the spatial distribution of *VFC* decreases gradually from southeast to northwest.

**Figure 5:**
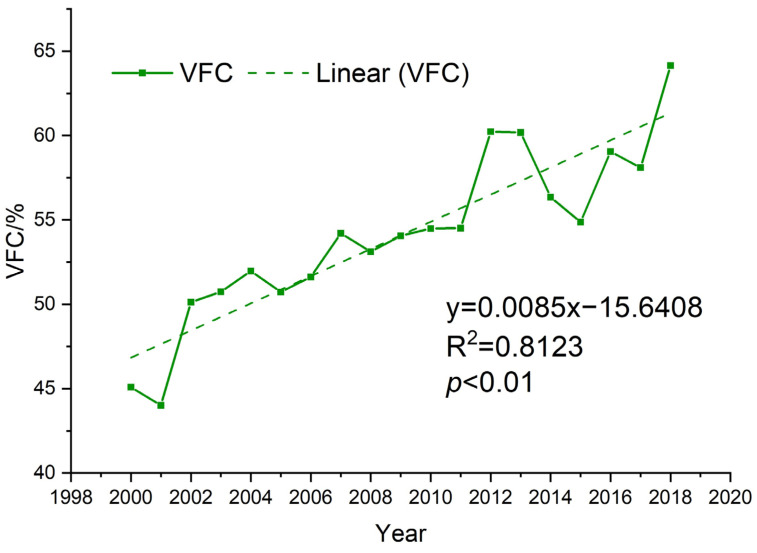
Temporal trends of *VFC* in the Loess Plateau from 2000 to2018.

**Figure 6:**
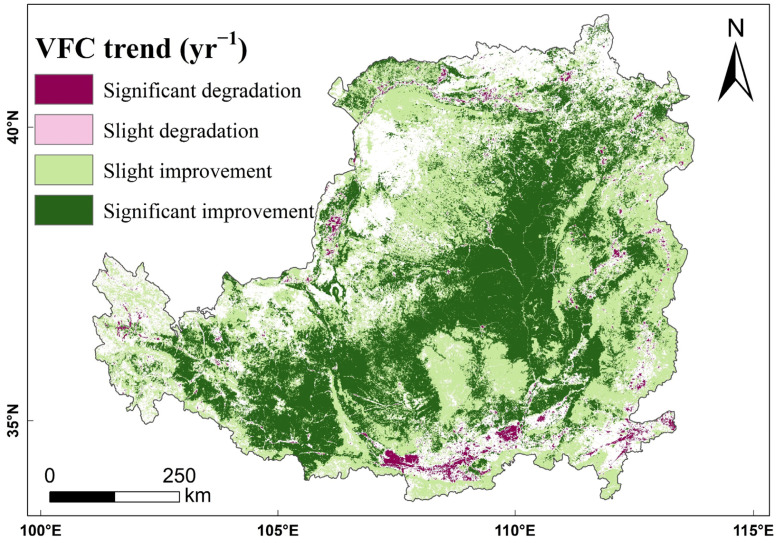
Spatial pattern of *NDVI* trends in the Loess Plateau from 2000 to 2018.

As shown in Figure 5, the overall *VFC* of the Loess Plateau showed a fluctuating increase (*p* < 0.01) from 2000 to 2018, from 45.09% in 2000 to 64.15% in 2018, with an improvement of 38.02% and an average annual growth rate of 2%. The trend analysis of the *VFC* from 2000 to 2018 shows that the *VFC* of 97.66% of the area of the Loess Plateau has been improved at the 5% significance level (Figure 6). Among the areas, 47.58% showed a significant improvement in *VFC*, mainly concentrated in the loess sorghum gully region and the loess hilly gully region; however, 50.07% of the areas showed a slight improvement in *VFC*, mainly concentrated in the earth–rocky mountain region and the sand and desert region. The *VFC* of the remaining 2.34% of the area showed a degradation trend, among which 2.32% of the area showed significant degradation, and 0.02% of the area showed slight degradation. The *VFC* degradation areas are mainly concentrated in the river valley plain region in the south of the Loess Plateau.

Further analysis showed that the low and medium-low *VFC* areas on the Loess Plateau decreased year by year from 2000 to 2018 (Table 2). Among these, the low *VFC* area decreased most significantly, with a reduction of 128,513.5 km^2^ in 18 years for a reduction rate of 19.79%. The medium-low *VFC* area decreased by 96,251.25 km^2^ in 18 years; the reduction rate was 14.82%. Meanwhile, the medium-high and high *VFC* areas are growing year by year. Among these, the growth in high *VFC* areas is the most significant, with a total growth of 133,713 km^2^ in 18 years and a growth rate of 20.59%. The medium-high *VFC* area had a total growth of 91,051.75 km^2^ in 18 years and growth rate of 14.02%.

### 3.3. Soil Loss

**Figure 7:**
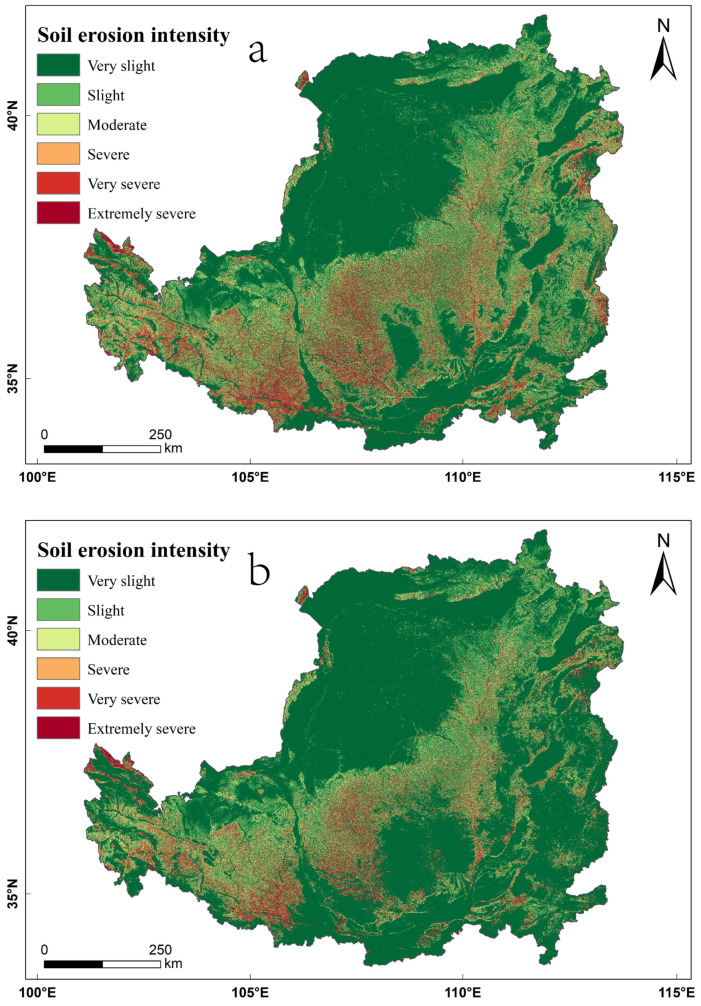
Distribution of soil erosion intensity in the Loess Plateau in (**a**) 2000 and (**b**) 2018.

In 2000 and 2018, the average soil erosion modulus of the Loess Plateau was 26.41 t·hm^−2^·yr^−1^ and 17.92 t·hm^−2^·yr^−1^, respectively, decreasing by 32.16% in that time. In total, soil erosion was reduced by 550 million tons over 18 years. The soil erosion statistics of the Loess Plateau in 2000 and 2018 revealed that the soil erosion intensity of the Loess Plateau is very slight, and the area of very slight erosion accounts for more than 62% of the total area (Table 3). From 2000 to 2018, the area of very slight erosion showed an increasing trend, with an area increase of 10.90% over 18 years (Table 3). The very slight erosion is mainly distributed spatially in the river valley plain region in the south of the Loess Plateau, the sand and desert region in the northwest and the irrigation agricultural region in the north, where the topography is flat, and erosion does not easily occur (Figure 7). From 2000 to 2018, the areas of the Loess Plateau with more than slight soil erosion showed a decreasing trend, and the reductions in the areas of slight, moderate, severe, very severe and extremely severe erosion were 3.15%, 2.35%, 1.70%, 2.15% and 1.55%, respectively, during the 18 years (Table 3). Areas with more than slight soil erosion were mainly distributed spatially in the loess sorghum gully region and the loess hill and gully region (Figure 7). These areas have broken terrain, thousands of gullies, large slopes, loose loess soils and poor erosion resistance, coupled with concentrated precipitation, resulting in severe soil erosion in the region.

To further understand the specific changes in erosion level, the ArcGIS 10.7 software was used to calculate the soil erosion intensity transfer matrix of the Loess Plateau from 2000 to 2018. As shown in Table 4, the stability rates of very slight, slight, moderate, severe, very severe and extremely severe erosion on the Loess Plateau from 2000 to 2018 were 99.49%, 61.56%, 59.10%, 52.99%, 54.48% and 53.74%, respectively. It can be concluded that as the erosion level increased, the rate of transformation between each soil erosion level also increased, and the soil erosion intensity in most areas changed from high to low. The percentages of area transferred to very slight erosion were 36.25%, 28.36%, 26.41%, 26.53% and 27.56% for the light, moderate, severe, very severe and extreme severe erosion intensity classes, respectively.

## 4. Discussion

### 4.1. The Impact of SLCP on Ecological Effects

**Figure 8:**
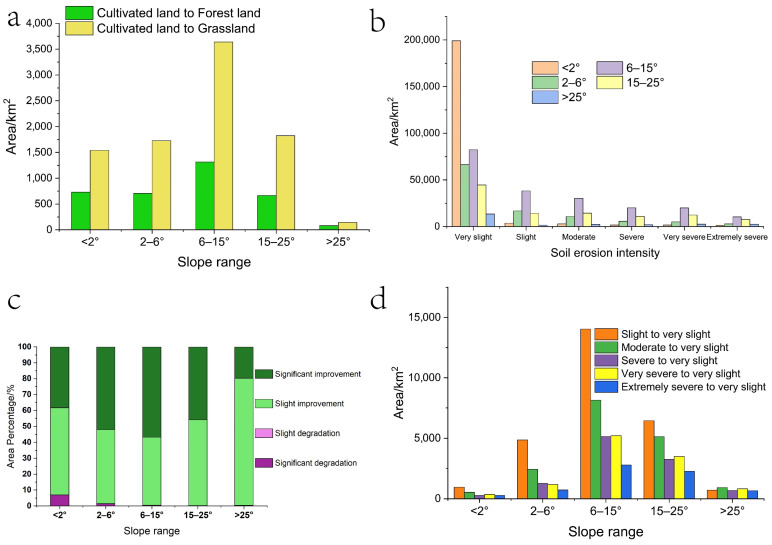
(**a**) Area of cultivated land converted to forest land and grassland for different slope ranges during 2000–2018. (**b**) Area of each soil erosion intensity class for different slope ranges in 2000. (**c**) Percentage of *VFC* change for different slope ranges during 2000–2018. (**d**) Area of each soil erosion intensity class above slight to very slight soil erosion for different slope ranges.

From 2000 to 2018, great ecological effects were achieved through the implementation of the SLCP on the Loess Plateau, mainly the significant conversion of sloping cultivated land to forest land and grassland areas, the improvements in *VFC* and the reduction in soil erosion, which are consistent with the conclusions obtained in previous studies [17,18,19,20,21,22,23,24,25,26,27,28,29,30,31,32,33,34,35,36,37,38,39,40,41,42]. According to the response of each ecological effect to the SLCP, it can be seen that the increase in forest land and grassland area in the Loess Plateau from 2000 to 2018 mainly came from the decrease in cultivated land area (Figure 3b), thus indicating that the SLCP was an important contributor to the change in cultivated land in the Loess Plateau, which is consistent with the conclusions reached by Chao et al. [17] and Li et al. [18]. Further analysis showed that the conversion of cultivated land to forest land and grassland was mainly concentrated in areas with slopes of 6–15°and 15–25° (Figure 8a), which is consistent with the conclusions of Zhang et al. [20] and Zhou et al. [21]. The reason for this is that before the SLCP was implemented, the areas with severe soil erosion on the Loess Plateau were mainly concentrated areas with slopes of 6–15° and 15–25° (Figure 7 and Figure 8b), so the 6–25° slope range was the core area for implementing the SLCP on the Loess Plateau. From 2000 to 2018, a total of 1981.34 km^2^ of cultivated land was converted to forest land, and a total of 5462.13 km^2^ of cultivated land was converted to grassland in the 6–25° slope range of the Loess Plateau, accounting for 56.61% and 61.57% of the total area of cultivated land converted to forest land and grassland, respectively. Moreover, in Figure 8c,d, it can be observed that from 2000 to 2018, the significant increase in *VFC* and the decrease in areas with more than slight soil erosion were also mainly concentrated in slopes of 6–15° and 15–25°, thus indicating that the Loess Plateau has seen significant increases in its areas of forest land and grassland through the implementation of the SLCP on the slopes of 6–15° and 15–25°, which thus promoted the improvements in *VFC* and curbed soil erosion. The 6–25° slope range is spatially concentrated in the loess sorghum gully region and the loess hilly gully region (Figure 1a), and the conversion of cultivated land to forest land and grassland on the Loess Plateau from 2000 to 2018 was mainly concentrated within this area (Figure 3a). The *VFC* has been significantly improved in this area (Figure 6), which has effectively curbed soil erosion (Figure 7b). This indicates that the 6–25° slope range was the core area for the implementation of the SLCP in the Loess Plateau and that the program has led to a significant improvement in the ecological environment of the Loess Plateau.

### 4.2. The Impact of Climatic Factors on the SLCP

**Figure 9:**
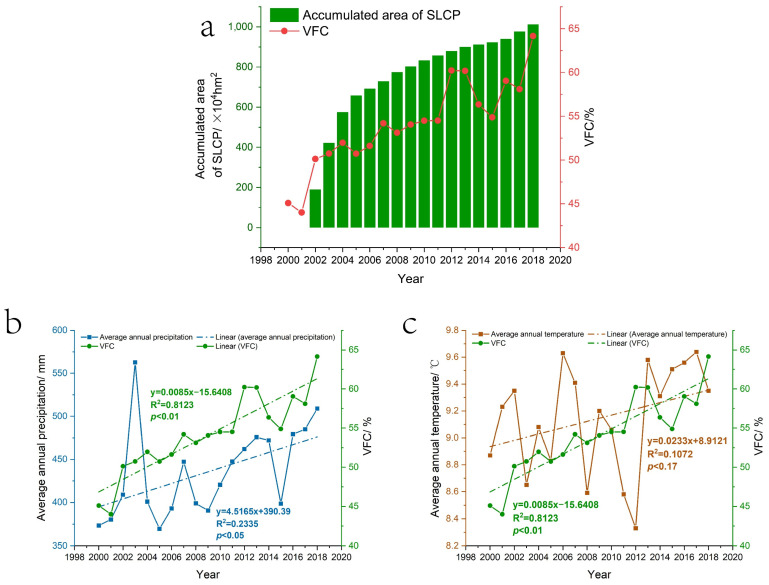
(**a**) Changes in *VFC* and the accumulated area of the SLCP on the Loess Plateau, 2000–2018; (**b**) changes in *VFC* and average annual precipitation on the Loess Plateau from 2000 to 2018; (**c**) changes in *VFC* and average annual temperature on the Loess Plateau from 2000 to 2018.

There is no doubt that the SLCP is an important factor that contributed to the improvement in the ecological environment of the Loess Plateau after 2000. As shown in Figure 9a, the Pearson’s correlation coefficient between the *VFC* of the Loess Plateau and the accumulated area of the SLCP in the Loess Plateau was as high as 0.888 (*p* < 0.01). However, vegetation changes are also influenced by climatic factors [66,67], such as temperature and precipitation, especially in the arid and semi-arid regions of the Loess Plateau, where the dry and wet conditions and the balance of water supply and demand have a decisive influence on vegetation growth [68,69]. The spatial distribution of *VFC* on the Loess Plateau is mainly decreasing from southeast to northwest (Figure 4), which is basically consistent with the spatial distribution of multi-year average precipitation on the Loess Plateau (Figure 1b). It can be concluded that precipitation was the dominant factor determining the spatial distribution of vegetation cover on the Loess Plateau. This is consistent with the conclusions of Sun et al. [66] and Xin et al. [70]. Secondly, as shown in Figure 9b,c, the annual average precipitation and temperature of the Loess Plateau showed a fluctuating upward trend from 2000 to 2018. The multi-year average value of precipitation was 435.55 mm, with an average annual growth rate of 1.91%, and the multi-year average value of temperature was 9.15 °C, with an average annual growth rate of 0.28%. These results indicate that the climate in the Loess Plateau region has tended to be warm and humid in the last 20 years, with an increase in temperature and precipitation, which is consistent with the conclusions of Li et al. [25] and Zheng et al. [29]. Previous studies have shown that climate warming and humidification would be beneficial for ecological restoration programs [71,72]. In Figure 9b, the variations in *VFC* and annual precipitation in the Loess Plateau are roughly consistent, and the Pearson’s correlation coefficient between them is 0.598 (*p* < 0.05), which shows that the improvements in the ecosystem of the Loess Plateau were affected by precipitation as well as the SLCP. In particular, in 2005 and 2015, the Loess Plateau *VFC* decreased due to a decrease in precipitation, while in 2007, the Loess Plateau *VFC* increased due to an increase in precipitation. A possible reason is that vegetation changes are very sensitive to precipitation [73], especially in arid and semi-arid regions, where precipitation has a significant positive relationship with improved vegetation [74,75,76]. Specifically for the Loess Plateau, increased precipitation can directly promote an increase in soil moisture in the arid and semi-arid areas of the plateau, which, in turn, promotes the growth of vegetation [77]. However, reduced precipitation may result in natural precipitation not meeting the recharge requirements of groundwater and deep soil water consumed by vegetation growth [78], thereby accelerating soil desiccation and limiting vegetation growth [79]. In Figure 9c, there is almost no consistency between *VFC* and the annual mean temperature change in the Loess Plateau, and no correlation between them could be obtained by calculation, thus indicating that vegetation growth on the Loess Plateau is less affected by temperature changes. However, there is still evidence that although precipitation has increased on the Loess Plateau, increased temperature accelerates water evaporation and exacerbates drought to some extent, making the increase in temperature detrimental to vegetation growth and ecosystem conditions [80,81].

## 5. Conclusions

The evaluation of the ecological effects of the SLCP, which is the largest ecological restoration program in the world, will not only provide guidance for the continued implementation of the SLCP in China but will also provide a reference for other countries in the world to evaluate the ecological effects of ecological restoration programs being implemented or to be implemented. The ecological effects created by the implementation of the SLCP are multifaceted; however, most previous scholars had only evaluated one aspect of its ecological effects in their studies, and a comprehensive evaluation of the ecological effects was lacking. This could have led to bias in policy makers’ understanding of the ecological effects of the SLCP. Based on this, we took the Loess Plateau, the core area for the implementation of the SLCP, as an example. Based on multi-source remote sensing data and with GIS technical support, we firstly evaluated the changes in land use, *VFC* and soil loss on the Loess Plateau after the implementation of the SLCP; analyzed the specific effects of the SLCP on these three ecological effects; and finally analyzed the influence of climatic factors on the SLCP. The conclusions are as follows.

(1) From 2000 to 2018, the implementation of the SLCP on the Loess Plateau achieved remarkable ecological effects. In terms of the temporal impact of the SLCP on ecological effects, the SLCP has led to increases in the areas of forest land and grassland on the Loess Plateau. The area of grassland generally showed a decreasing trend, but the area of grassland showed a significant increasing trend in the core area of the SLCP. The increases in forest land and grassland consequently led to an increase in *VFC* and a decrease in soil erosion. In terms of the spatial impact of the SLCP on ecological effects, the slopes of 6–15° and 15–25° are the core areas for the implementation of the SLCP on the Loess Plateau, and the areas where each ecological effect was significantly improved are also concentrated there, mainly in the loess sorghum gully region and the loess hilly gully region.
(2) Ecosystem improvements in the Loess Plateau from 2000 to 2018 were influenced by both the SLCP and climate change. The warming and humidification of the Loess Plateau climate tended to contribute, to a certain extent, to the implementation of the SLCP; precipitation had a greater impact on the vegetation changes on the Loess Plateau. At the same time, precipitation is also a dominant factor determining the spatial differentiation of vegetation on the Loess Plateau.

## Figures and Tables

**Table 1:** Statistical table of the percentage and area of land use types in the Loess Plateau (unit: km^2^).

Land Use Types	2000		2018		Change Rate(%/a)
	Area	Proportion	Area	Proportion	2000–2018
Cultivated land	217,365.31	33.48%	207,595.62	31.97%	−0.25
Forest land	104,521.47	16.10%	109,614.19	16.88%	0.27
Grass land	258,855.74	39.87%	256,568.11	39.51%	0.05
Water body	9178.82	1.41%	9120.42	1.40%	0.04
Built-up land	16,072.09	2.48%	26,626.24	4.10%	3.65
Bare land	43,300.14	6.67%	39,769.35	6.13%	0.45

**Table 2:** Area statistics and changes in *VFC* classes in Loess Plateau from 2000 to 2018 (unit: km^2^).

*VFC* Class	2000		2018		Change Rate (%)
	Area	Proportion	Area	Proportion	2000–2018
Low *VFC*	159,619	24.58%	31,105.5	4.79%	−19.79
Medium-low *VFC*	224,146.5	34.52%	127,895.25	19.70%	−14.82
Medium-high *VFC*	172,117	26.51%	263,168.75	40.53%	14.02
High *VFC*	93,394.75	14.38%	227,107.75	34.98%	20.59

**Table 3:** Area statistics and changes in the soil erosion intensity class in the Loess Plateau from 2000 to 2018 (unit: km^2^).

Erosion Intensity	2000		2018		Change Rate (%)
	Area	Proportion	Area	Proportion	2000–2018
Very slight	405,692	62.68%	476,267.75	73.59%	10.90
Slight	74,540	11.52%	54,129	8.36%	−3.15
Moderate	60,586	9.36%	45,374.75	7.01%	−2.35
Severe	40,192.5	6.21%	29,211.5	4.51%	−1.70
Very severe	41,733.5	6.45%	27,808	4.30%	−2.15
Extremely severe	24,485.25	3.78%	14,439.25	2.23%	−1.55

**Table 4:** Soil erosion intensity transfer rate on the Loess Plateau, 2000–2018.

Erosion Intensity	Very Slight	Slight	Moderate	Severe	Very Severe	Extremely Severe
Very slight	99.49%	0.18%	0.21%	0.06%	0.04%	0.02%
Slight	36.25%	61.56%	0.89%	0.81%	0.40%	0.08%
Moderate	28.36%	10.67%	59.10%	0.75%	0.64%	0.48%
Severe	26.41%	1.25%	17.67%	52.99%	0.91%	0.76%
Very severe	26.53%	1.10%	1.61%	15.03%	54.48%	1.25%
Extremely severe	27.56%	0.43%	1.20%	1.38%	15.69%	53.74%

## Data Availability

The data used in this study are described in detail in Section 2.3. The data sources and all data are available for download through the URL link provided in that section; alternatively, please contact the author of this article.
